# The influence path of community green exposure index on activity behavior under multi-dimensional spatial perception

**DOI:** 10.3389/fpubh.2023.1243838

**Published:** 2023-10-02

**Authors:** Lingyu Zheng, Yixue Zhao, Ran Duan, Wanting Yang, Zhigang Wang, Jiafu Su

**Affiliations:** ^1^Art College, Chongqing Technology and Business University, Chongqing, China; ^2^Faculty of Smart Urban Design, Chongqing Jianzhu College, Chongqing, China; ^3^International College, Krirk University, Bangkok, Thailand

**Keywords:** spatial perception, activity behavior, community green space, normalized difference vegetation index, green view index

## Abstract

The purpose of this research is to reveal the internal relationship among community green space, space perception, and activity behavior response to supplement the lack of research results on the binary relationship between green space and behavior. Nine residential community green spaces and 398 residents were selected as the research objects. Thematic clustering and factor identification were used to determine the spatial dimensions of community green space that residents were concerned about. The analysis of the green exposure index, spatial perception evaluation, and activity behavior survey were combined to determine the influence of the green exposure index on spatial perception and activity behavior and its internal correlation path. According to research data, the community green view index (GVI) and normalized difference vegetation index (NDVI) negatively affected the perception factor, while the perception factor positively affected the activity frequency. The SEM model shows that the green exposure index stimulated activity behavior through the intermediate effect of the internal perception path of perceived landscape quality, perceived workability, and perceived accessibility. Spatial perception as the basis of the instantaneous emotional reaction process may affect people's choices for activities but be unable to extend the duration of the activities. The internal association among community green space, spatial perception, and physical activity behavior develops on the basis of spatial patterns at certain scales. This study provides a theoretical basis for understanding the spatial experience and residents' behavioral needs, evaluating the quality of urban green space scientifically, and promoting the optimization of community green space structure.

## 1. Introduction

Human beings, going through evolution over millions of years, have formed a psychological mechanism for adapting to the natural environment. The physical attributes of the natural environment lead to aesthetic preferences and emotional responses through perceptual filters, which then affect psychological emotions and cognition (1, 2), relieve mental fatigue, and balance physiological functions, finally contributing to the stimulation of behaviors or functions so as to promote health and survival adaptation (3, 4). As a sensory interaction process to obtain information about the natural environment, “perception” is the psychological foundation as well as the determinant of individual decision-making across time and space (5). As the representative subject of urban natural environment, urban green space carries four potential ways to improve the ecological environment, restore physiological capacity, promote physical activity, and improve social interaction with public health (6). Empirical research has found that the complexity and multi-dimensional nature of urban green spaces may lead to different psychological reactions and behavioral stimuli, which in turn affect physical activity levels (7, 8). Therefore, compared with the objective geographical space characteristics, the subjective perceptual attributes of urban green space exert much more empirical value for residents to participate in outdoor activities and maintain their physical health.

Green space exposure assessment is generally considered a scientific evaluation method for studying urban green space and population health. It consists of two major evaluation indices, which are the two-dimensional ground greening evaluation index normalized difference vegetation index (NDVI) and the facade space greening evaluation index green view index (GVI) (9). By capturing the growth potential and increment of ground vegetation through satellite images, NDVI can reflect the density of above-ground green vegetation in a relatively accurate manner, and it is widely applied to the classification of urban land cover types, the assessment of urban ecological environment quality, and the research on the relationship between urban green space and health (10, 11). GVI refers to the proportion of green parts in the field of vision (12), and it is regarded as an evaluation index to reveal the perception preference in human settlements and measure urban greening construction. In recent years, NDVI and GVI supplement each other in two different spatial dimensions (ground two-dimensional scene and street three-dimensional scene) so as to achieve a comprehensive evaluation of urban greening quality and green spatial perceived experience, thereby being regarded as two indicators most applicable to the empirical analysis of the relationship between urban green space and physical activity behavior as well as epidemiological health results (13).

Community green space in China as a type of urban green space is highly relied on by residents in daily life to provide a safer, accessible, and attractive environment in the neighborhood. However, it may cause less daily use and participation in physical activities for residents due to a series of problems such as unreasonable space planning, excessive greening, unscientific plant configuration, or poor maintenance and management (14). From a practical perspective, community green space was planned and designed to mainly meet the amenity value and ecological environment benefits, providing recreational places and satisfying space experience as the least prior functions. From the theoretical perspective, most relevant studies emphasize the binary relationship between green space and individual psychology (5), behavior, or health, ignoring the intermediate role of visual perception interaction between humans and the environment, and it is difficult to identify the characteristics of green space that residents truly prefer and experience comfort. However, some studies have pointed out that there are biopsychosocial pathways between community green space exposure and health, indicating that there are multiple relationships between green space and health or behavior. This study focused on the intermediate role of visual perception and proposed a hypothetical path of the green exposure index affecting activity behavior through spatial perception. A regression analysis and a structural equation model were used to reveal the effect of the green exposure index based on multidimensional perception of activity behavior and its internal relationship. It is hoped that this study can provide theoretical support for accurate community planning and decision-making as well as the creation of healthy community life circles.

## 2. Methodology

### 2.1. Study area and data

**Figure 1:**
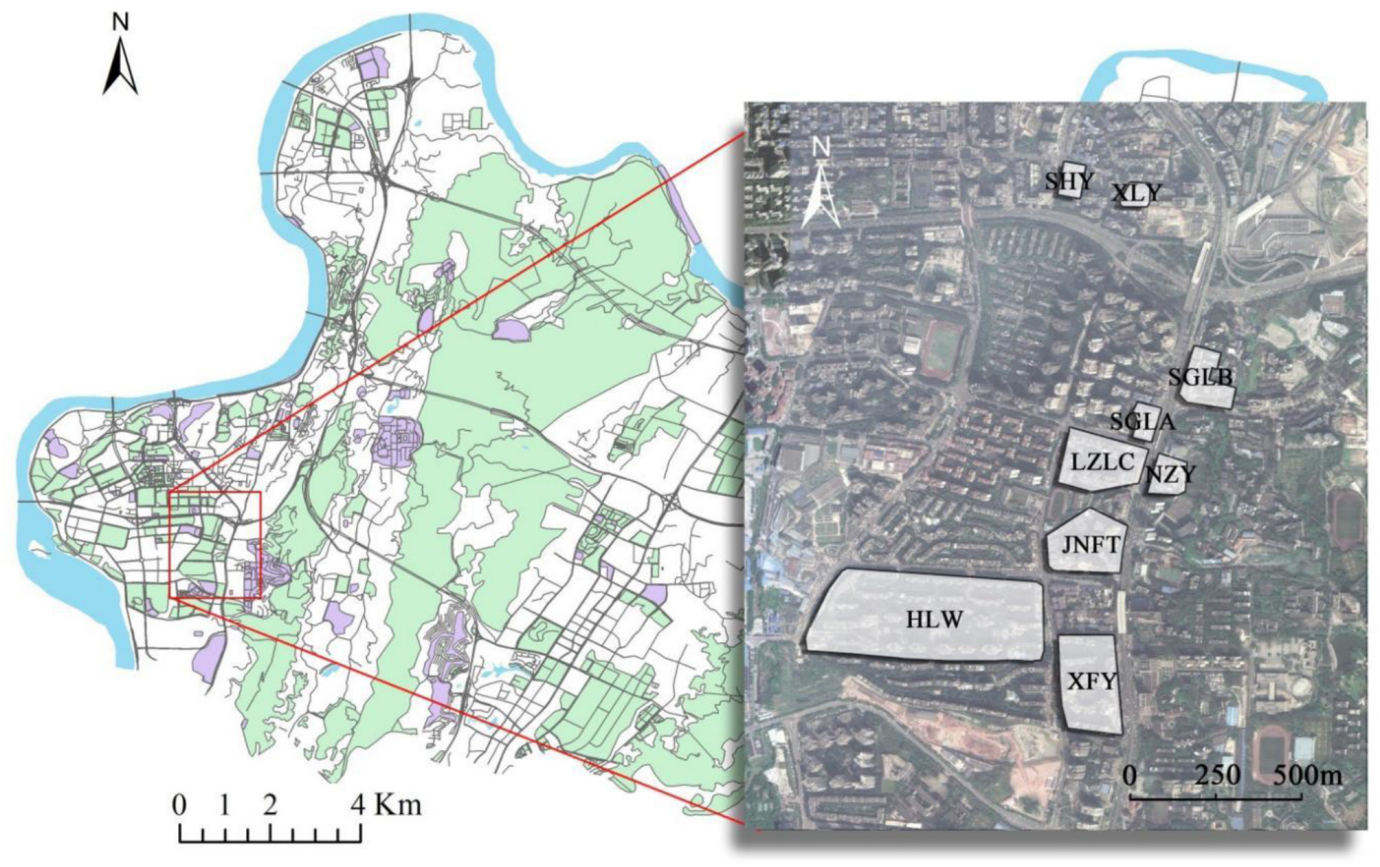
The bottom map is the administrative area of Nan'an District, Chongqing, where the research community is located. A zoom is made to highlight the spatial boundaries of the research community.

**Figure 2:**
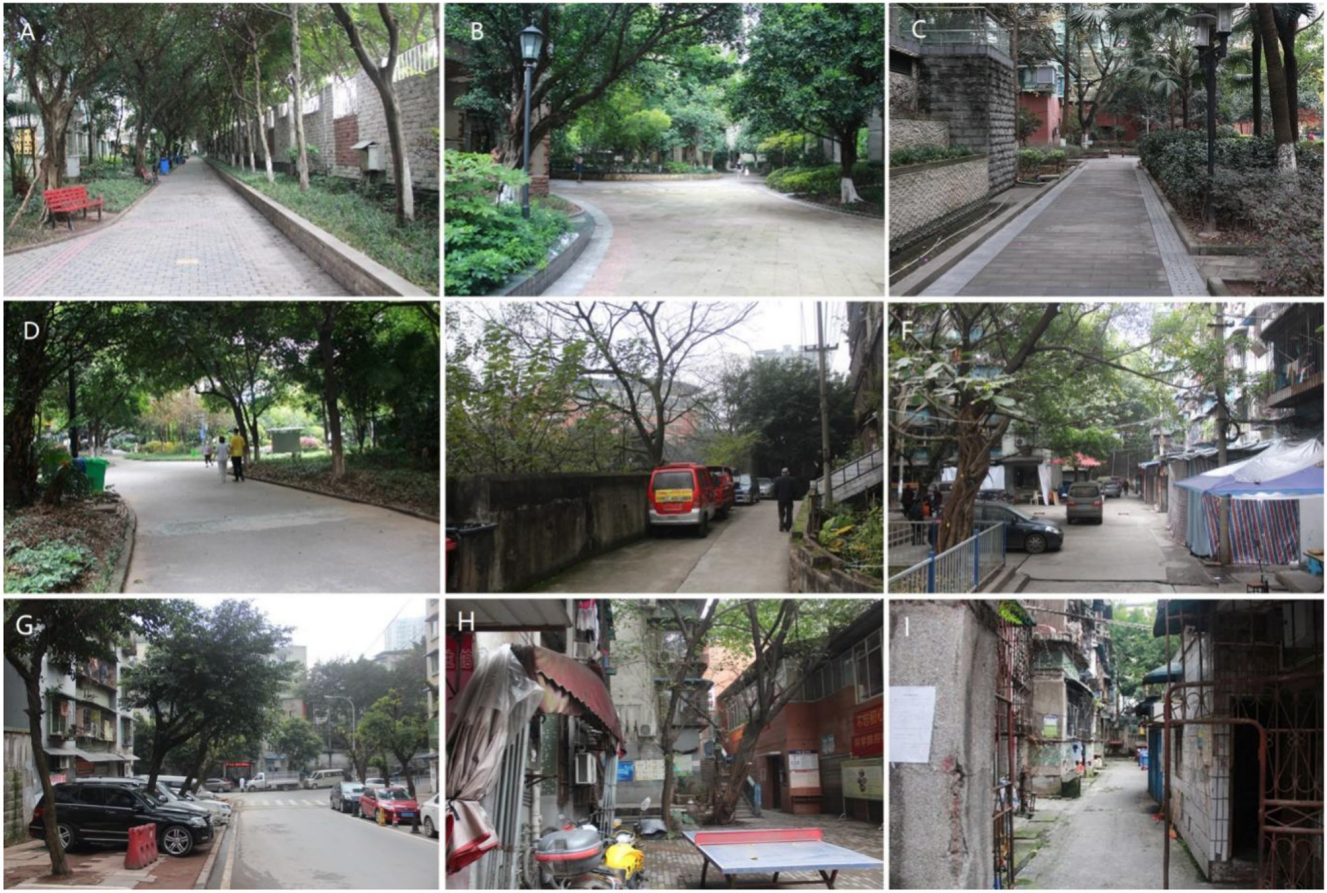
Realistic pictures of the research community. **(A)** Huilongwan community (HLW); **(B)** Jiangnan Fenting community (JNFT); **(C)** Luzhou Longcheng community (LZLC); **(D)** Xuefuyuan community (XFY); **(E)** Nanzhongyuan community (NZY); **(F)** Sanheyuan community (SHY); **(G)** Xinglongyuan community (XLY); **(H)** Sigongli A community (SGLA); and **(I)** Sigongli B community (SGLB).

Investigation destinations are located on Haitangxi Street, Nan'an District, Chongqing, China. The research communities include nine residential communities (Figures 1, 2): Huilongwan community (HLW), Jiangnan Fenting community (JNFT), Luzhou Longcheng community (LZLC), Nanzhongyuan community (NZY), Xuefuyuan community (XFY), Sanheyuan community (SHY), Xinglongyuan community (XLY), Sigongli A community (SGLA), and Sigongli B community (SGLB). Housing properties include three types: commercial housing (CH), unit community (UC), and security housing (SH). The investigation was conducted in June with the best landscape effect and minimum external factors, and the whole process was conducted on cloudy days to avoid the potential impact of weather on personal subjective feelings. Older adults with no allergic history, aged 50–70 years, with certain thinking abilities as well as language skills, were chosen as respondents. To avoid the exclusion or avoidance of the respondents to the investigation process, structured interviews and questionnaire surveys were performed in the research community after random sampling in the main form of a team survey involving professional investigators, property management personnel, and undergraduate students.

### 2.2. Problem measurement and questionnaire design

To ensure the reliability, validity, and accuracy of the expression content of research variables, the research systematically reviewed the domestic and foreign literature, and then thematic cluster analysis was taken on green spatial perception variables at the neighborhood scale. Through pre-survey, three aspects of community residents were obtained: (1) the overall feeling of community green space and the experience value of site-specific information; (2) views and opinions on the role and management of green space; and (3) understanding of the function, use, and importance of green space. Through the collation of information, the most commonly used descriptive word by the residents and the space experience measuring variables most valuable for community green space were selected. Eight perception measuring items initially obtained were then used for exploratory factor analysis, and three perception factors with the highest degree of association were extracted. According to variable attributes, these three perception factors were named perceived landscape quality (Q), perceived accessibility (A), and perceived workability (U), respectively (Table 1). Among them, Q included two measuring items, which were naturalness (Q1) and aesthetics (Q2); U included three measuring items, which were safety (A1), friendliness (A2), and connectivity (A3); and U included three measuring items, which were cleanliness (U1), facility integrity (U2), and functionality (U3). Each measuring item represented a measuring variable containing several measuring evaluation problems. Finally, a perception evaluation system was established, and a comprehensive perception evaluation of community green space was revealed from different perception dimensions.

**Table 1:** Community green spatial perception evaluation index system.

**Factor no**.	**Perception factor**	**Measuring item no**.	**Perception measuring items**	**Measuring problem**
Q	Perceived landscape quality	Q1	Naturalness	The naturalness of plant landscape; the diversity of vegetation species; the variety of topography.
		Q2	Aesthetics	The aesthetics of design elements; tranquil landscape experience.
A	Perceived accessibility	A1	Safety	Sense of security; sense of shelter.
		A2	Friendliness	Freedom to experience; spatial sense of belonging.
		A3	Connectivity	The difficulty level of entering and accessing to green space.
U	Perceived workability	U1	Cleanliness	Care and maintenance for green space; vegetation trimming and conservation.
		U2	Facility integrity	Proportion of space for activity facilities; the variety of activity facilities.
		U3	Functionality	The usability of dynamic and sedentary activities.

The “community green spatial perception and activity behavior” questionnaire was designed, and three aspects were contained: (1) socio-demographic characteristics of the respondents; (2) community green spatial perception evaluation; and (3) green space activity evaluation of recreational physical activity behavior. The community green spatial perception evaluation in the questionnaire adopted the 5-grade Likert scoring system for the above-constructed evaluation system. This study mainly focused on outdoor physical exercise, which was one of the four major types of physical activity, excluding leisure time chatting or sedentary activities. The measuring items targeting recreational physical activity behavior in green space activity were identified as activity time (PT) and activity frequency (PF) of recreational physical activity, namely the outdoor physical exercise in an average week. The evaluation score was given according to the length and frequency of time, in which PT referred to the average use time of green space in each activity (PT ≤10 min, 1 score; 11–20 min, 2 scores; 21–30 min, 3 scores; and ≥30 min, 4 scores); PF referred to the number of times of green space activities in an average week (PF = 0, 1 score; 1–2, 2 scores; 3–6, 3 scores; 7–10, 4 scores; and over 10, 5 scores).

### 2.3. Determination of the green exposure index

Normalized difference vegetation index (NDVI): The satellite remote sensing image of the research community (precision: 10 × 10 m) was visually interpreted using the ArcMap 10.2 software, and information including the location, type, and area information of the green space boundaries, roads, and buildings in the research community was extracted. The remote sensing image went through spectral analysis using ENVI5.6, and the NDVI value of the research community was calculated by the ratio of the difference between the values of the near-infrared band and the visible red band and the sum of the values of these two bands (value range: 0–1).

Green view index (GVI): By using mobile GPS (Google Map App), equidistant sample points (30 m each) were set along the walking path of the main roads in the research community, and then a camera (Canon 600D) was placed on the tripod at each sample point. At a horizontal angle of view, 1.5 m from the ground, panoramic images in four directions were captured at each sample point. With reference to the general definition and measuring method in the Guide to the Investigation and Research of Green Visual Ratio (15, 16), Adobe Photoshop CS6 was applied to correct the image and extract the contour of the green part, including plant leaves and water bodies while excluding branches and blocked parts. The calculation formula is: GVI = green part area/total photo area × 100%. The GVI value and the average GVI value of the research community were obtained.

### 2.4. Statistics and analysis

Excel and IBM SPSS Statistics 25.0 software were applied to collect and analyze the survey data of community green exposure index, spatial perception, and activity behavior, following the relevant associated analysis. Analysis methods include descriptive analysis to uncover the basic characteristics of the research object and the research community. Exploratory factor analysis of the perception measure item extracts the three perception factors with the highest correlation degree according to the factor load and determines the perception dimension of community green space. Mean comparison analysis and regression analysis verify the impact of the green exposure index on community green spatial perception and activity behavior, and to a certain extent indicate that spatial perception may participate in the influence path as an intermediate factor. According to the regression results and research hypotheses, the AMOS structural equation model was constructed to explain the action path of multi-dimensional spatial perception in the process of green exposure index inducing activity behavior and reveal the internal correlation path of green exposure index, multi-dimensional spatial perception, and activity behavior.

## 3. Results and analysis

### 3.1. Description of basic information

By conducting a related investigation, complete and valid data on 398 respondents in total in the nine residential communities were obtained (Table 2). In this table, the CH communities (HLW, JNFT, LZLC, and NZY), together with the UC community XFY, was built in this century. The SH communities (SHY, XLY, SGLB, and SGLA) were built from the 1970s and 1980s to the beginning of this century, covering an area that was generally smaller than the CH communities and the UC community. It was observed from space information analysis that the GVI value of the research community was concentrated between 10 and 60%, while the NDVI value was concentrated between 0.20 and 0.60. Both the GVI and NDVI of the SH communities were lower than those of the CH communities and the UC community.

**Table 2:** The information list of the research community.

**Community name**	**Housing attribute**	**Time of construction/year**	**Floor space/m^2^**	**GVI**	**NDVI**	**Number of respondents**
HLW	CH	2007	167,500	54.0%	0.521	52
JNFT	CH	2004	29,700	52.9%	0.407	47
LZLC	CH	2004	28,600	53.0%	0.337	54
NZY	CH	2000	10,400	26.0%	0.228	19
XFY	UC	2001	45,800	55.3%	0.419	53
SHY	SH	2000	5,700	19.5%	0.397	47
XLY	SH	2000	3,400	32.3%	0.382	53
SGLB	SH	1980	16,200	9.5%	0.194	48
SGLA	SH	1970	4,900	12.7%	0.205	25

### 3.2. Analysis of community green spatial perception and activity behavior

Perception-measuring items with more than four scores are considered of significance or with positive value. According to the comparison analysis of spatial perceived importance attribution and activity behavior evaluation (Table 3), there was a certain relationship between the proportion of residents with perceived importance and the mean value of activity behavior evaluation. In other words, it was indicated that the higher the number of perception-measuring items with positive values (the proportion of residents was over 50%), the higher the mean value of activity behavior evaluation, especially the PF value. For example, the proportion of importance of eight perceived measuring items in the XLY community was over 50%, and the mean value of evaluation of PT and PA was higher than other communities, while in the XFU community, only two perceived measuring items had an importance of more than 50%, along with the lowest mean value of evaluation of PT and PA. The above results indicated that there may exist a correlation between space-perceived measuring items and activity behavior in the research community.

**Table 3:** Spatial perception importance attribution and activity behavior evaluation analysis.

**Community name**	**Proportion of people evaluating spatial perception importance (%)**								**Mean value of the activity behavior evaluation**	
			**A1**	**A2**	**A3**	**U1**	**U2**	**U3**	**PT**	**PA**
HLW	16.7	14.8	66.7	72.2	42.8	5.6	11.1	29.7	3.20	3.96
JNFT	17.0	29.8	51.1	65.9	35.2	8.5	0	57.4	3.32	3.98
LZLC	18.5	29.7	63.0	59.3	59.3	9.3	25.9	33.4	2.44	3.48
NZY	15.8	21.1	57.9	84.3	79.0	5.3	15.8	52.7	3.11	4.05
XFY	16.4	10.9	76.4	90.0	32.7	5.5	7.3	25.5	2.85	3.36
SHY	74.4	67.9	74.4	84.5	80.9	23.4	44.7	55.3	3.17	3.79
XLY	100	83.6	89.1	72.7	87.2	61.8	63.7	98.2	3.69	4.15
SGLB	65.3	69.3	77.6	67.3	98.0	26.5	65.3	64.3	1.59	4.51
SGLA	52.0	36.0	64.0	64.0	28.0	24.0	12.0	80.0	3.20	3.84

According to the community green exposure index and linear regression analysis (Table 4), GVI and NDVI negatively affected the three perception factors, among which perceived landscape quality was most affected. Specifically, GVI exerted a more prominent impact on the seven perception factors except the friendliness factor (*P* < 0.05). NDVI had no obvious impact on friendliness and security factors but exerted a significantly negative impact on the rest of the six perception measuring items (*P* < 0.05). The results of community spatial perception and activity behavior regression analysis (Table 5) indicated that the three perception factors and their measuring items all positively affected the activity frequency, except that the workability and cleanliness factors all positively affected the activity time.

**Table 4:** Unitary regression analysis of community green exposure index and spatial perception.

**Perception measuring item**	**GVI**			**NDVI**		
	***B***	***F*-value**	***P***	***B***	***F*-value**	***P***
Perceived landscape quality	−0.500	134.108	0.000	−0.274	32.706	0.000
Naturalness	−0.504	136.904	0.000	−0.262	29.586	0.000
Aesthetics	−0.432	92.502	0.000	−0.252	27.330	0.000
Perceived accessibility	−0.280	34.401	0.000	−0.168	11.636	0.001
Safety	−0.120	5.877	0.016	−0.059	1.409	0.236
Friendliness	−0.080	2.619	0.106	−0.28	0.322	0.571
Connectivity	−0.369	63.620	0.000	−0.249	26.557	0.000
Perceived workability	−0.459	107.450	0.000	−0.274	32.708	0.000
Cleanliness	−0.319	45.510	0.000	−0.191	15.280	0.000
Facility integrity	−0.404	78.783	0.000	−0.207	17.982	0.000
Functionality	−0.433	93.025	0.000	−0.297	38.908	0.000

**Table 5:** Unitary regression analysis of community spatial perception and activity behavior.

**Perception factor/measuring item**	**Activity time**			**Activity frequency**		
	***B***	***F*-value**	***P***	***B***	***F*-value**	***P***
Perceived landscape quality	0.084	2.837	0.093	0.148	9.080	0.003
Naturalness	0.093	3.489	0.062	0.136	7.607	0.006
Aesthetics	0.064	1.637	0.202	0.142	8.332	0.004
Perceived accessibility	0.071	2.014	0.157	0.235	23.457	0.000
Safety	0.081	2.640	0.105	0.179	13.318	0.000
Friendliness	0.021	0.180	0.672	0.114	5.348	0.021
Connectivity	0.048	0.941	0.340	0.199	16.606	0.000
Perceived workability	0.114	5.265	0.022	0.238	24.226	0.000
Cleanliness	0.143	8.410	0.004	0.165	11.275	0.001
Facility integrity	0.086	3.007	0.084	0.202	17.139	0.000
Functionality	0.055	1.244	0.265	0.234	23.347	0.000

### 3.3. Analysis of the association path between green exposure index and activity behavior based on spatial perception

**Figure 3:**
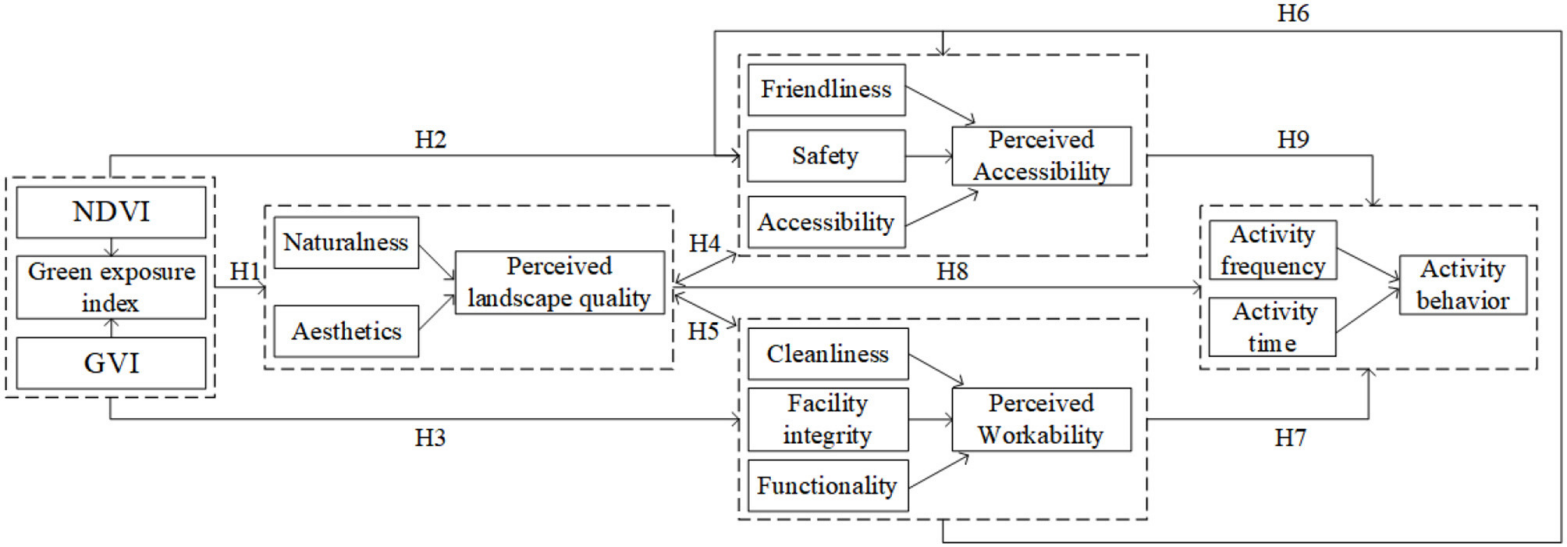
The concept model.

The statistical analysis of the above data suggested that the community green exposure index may be associated with activity behavior through spatial perception factors. To clarify the relationship among the three factors, the following hypotheses were proposed: (1) the green exposure index directly affected the spatial perception factors and caused activity behavior and (2) the perception factors were internally involved in the process of the green exposure index affecting activity behavior through spatial perception. Based on the above hypotheses, a structural equation model (Figure 3) was constructed, and then activity behavior (activity frequency and activity time) was included in the model as a latent variable to be examined. Though the goodness-of-fit of the hypothesis model was overall high, perception factors exerted an inconspicuous impact on the activity behavior (the results are not shown). The model was rebuilt after excluding the activity time, which led to a hybrid model based on activity frequency response. It was observed that the fitted values of major testing indicators were all within the recommended range (Table 6), which indicated that the hypothesis model could match the statistics with high goodness-of-fit. The above data supported the hypotheses proposed by the model.

**Table 6:** The value of fit indices of the structural equation model.

**Fit indices**	**Recommended value**	**Fitted value**
χ^2^/df	<3.0	2.290
GFI	>0.9	0.964
AGFI	>0.8	0.932
RMSEA	<0.08	0.057
NFI	>0.9	0.953
IFI	>0.9	0.973
CFI	>0.9	0.973

**Figure 4:**
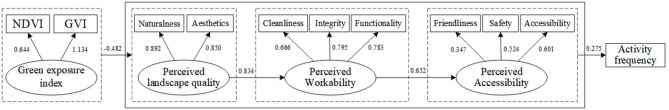
The realistic hypothesis model.

In the hypothesis-testing results of the structural equation model (Table 7), the results of hypothesis paths H1, H3, H5, H6, and H9 passed the *t*-test (*P* ≤ 0.05). It demonstrated the successful establishment of the hypothesis paths and indicated that there existed some influence rule between the green exposure index and the activity frequency of the residents under the intermediate effect of perception factors (perceived landscape quality, perceived workability, and perceived accessibility). The realistic hypothesis model was obtained after sorting out related statistics (Figure 4), in which the green exposure index first negatively affected the perceived landscape quality and then positively affected the perceived workability and accessibility, respectively, finally exerting a positive impact on the activity frequency. This model demonstrated that the green exposure index could indirectly affect the frequency of outdoor physical activity among residents through the intermediate function of perception factors. Moreover, there was a close internal correlation and response path among the three dimensions representing perception factors (perceived landscape quality, perceived workability, and perceived accessibility).

**Table 7:** Hypothesis-testing results of the structural equation model.

**Path no**.	**Hypothetical relationship**	**Normalized path coefficient**	***t-*value**	**Hypothesis supported or not**
H1	Green exposure index ↔ perceived landscape quality	−0.482^***^	−8.796	Yes
H3	Green exposure index ↔ perceived workability	−0.459^***^	−7.743	Yes
H5	Perceived landscape quality ↔ perceived workability	0.834^***^	9.283	Yes
H0	Green exposure index → activity frequency	0.041	−0.881	No
H2	Green exposure index → perceived accessibility	−0.111	−1.765	No
H4	Perceived landscape quality → perceived accessibility	−0.004	0.983	No
H6	Perceived workability → perceived accessibility	0.652^***^	3.544	Yes
H7	Perceived workability → activity frequency	0.273	1.705	No
H8	Perceived landscape quality → activity frequency	−0.232	−1.786	No
H9	Perceived accessibility → activity frequency	0.275^**^	2.380	Yes

Significance ^***^P < 0.001; ^**^P < 0.01.

## 4. Discussion

### 4.1. Identification and description of urban green spatial perception factors

Since the 1970s, environmental psychologists have proposed that natural landscape perception can significantly improve emotions and affect behavior. The natural environment information forms aesthetic preferences through sensory contact and stimulates emotional responses and neurophysiological activities. Therefore, perception is seen as an intermediate process between the natural environment and the behavioral response (17–19). As the representative subject of the natural environment in urban cities, urban green space exerts a profound impact on modern human space experience, daily behavior, and physical and mental health (20). Through relevant literature, it is found that the perceptual attributes of urban green space can be divided into two dimensions: one is perceived landscape quality attribute which focuses on the universality and significance of natural landscape aesthetics (21–23), covering a wide range of characteristic indicators including complexity, aesthetics, naturalness, culture and history, openness, serenity, comfort property, and species diversity (24–27), and the other is perceived use attribute that emphasizes on green space ecosystem services and management functions (28), generally targeting green space units such as parkland or neighborhood community green space and including various characteristic indicators such as cleanliness, site and facility characteristics, safety, convenience, functionality, walking accessibility, and social nature (29, 30). Although some perception variables were sorted out through investigation and regression analysis, there were two major problems in previous studies. First, empirical studies generally discussed the binary relationship between physical attributes and behaviors of green space, ignoring the quantitative relationship between the intermediate roles of perception variables. Second, few analyses revealed the internal relationship between perception variables. It is generally believed that perception variables are in the same perception dimension, ignoring the multi-dimensions of human attention to landscape and the relationship between different dimensions. This research proposes the hypothesis that the green exposure index affects activity behavior through spatial perception, highlighting the significance of multi-dimensional spatial perception. The research method is a supplement to the binary relationship between green exposure and activity behavior, which has a certain exploratory nature.

### 4.2. Influence of green exposure index on spatial perception and activity behavior

In this study, GVI and NDVI constitute important green vegetation indices from two dimensions to facade space, and they are considered measuring indices that practically and effectively examine the relationship between three-dimensional perceived green quantity and population health. According to the survey of the Ministry of Land, Infrastructure, Transport and Tourism of Japan, a GVI of more than 25% can render a better view of greening and make people feel visually comfortable. Moreover, a large number of scholars have found that GVI between 30 and 50% can produce a nice landscape, relieve fatigue, or gather crowds of people, and there is an inverted *U*-shaped relationship between space satisfaction, pressure recovery, and GVI. That is to say, as the GVI value increases, the landscape satisfaction may be reversed and the pressure recovery may be hindered (31, 32). In addition, in neighborhood spaces with medium and high levels of NDVI, residents tend to spend more time taking recreational physical activities in summer, but increased NDVI values may also reversely reduce the level of walking or cycling (33). In this study, the green exposure index had a negative impact on all the spatial perception measuring items, and the higher the GVI and NDVI values were, the lower the community perception factor evaluation was, which indicated that people had a higher degree of preference or sensitivity to community green space with moderate GVI and NDVI values. When the greening degree was too high, vegetation became so dense that people's views may be blocked, which may adversely affect the three perception dimensions as well as activity behavior.

### 4.3. The internal association path of green spatial perception on activity behavior

Psychological evolution theory suggests that natural environment information is the first to interact through visual perception in contrast to other sensory perceptions. The overall structure, depth characteristics, and scene categories of the natural landscape will directly affect individual visual perceptual attributes and then activate adaptive behaviors or functions through aesthetic preference, cognitive advantages and disadvantages, and behavioral motivation, and finally forming an emotional response process in the natural landscape. Therefore, GVI as a green visual index, in contrast to the two-dimensional vegetation cover evaluation index NDVI, may exert a more prominent impact on the community's green spatial perception factors evaluation and activity behavior as it reflects the overall characteristics of green space and space sensory experience with the fastest speed (34). On the other hand, brain science experts and human geographers have found that emotions are stimulated by the external environment and that emotions drive behavior. As two major expressions of emotions in man–land relationships, love and fear dominate people's emotions, making them go after advantages and avoid disadvantages (35). The unity of opposites of love and fear constitutes the basis for people to understand the dialectical relationship between man and space, so the brain mechanism behind the adaptive behavior of “going after advantages and avoiding disadvantages” constitutes the basic process for organisms to adapt to the environment (36). In this study, the green exposure index affected activity behavior through the intermediate effect of the internal perception path of perceived landscape quality, perceived workability, and perceived accessibility, indicating that the three perception dimensions, under the emotional response framework, may be regarded as intermediate response factors in the process of aesthetic preference to cognitive advantages and disadvantages; that is, the community green space environment affected subjective cognition and behavioral motivation and finally motivated activity behavior through sensory interaction to stimulate the responsive process of the overall quality preference evaluation (at the natural environment level), landscape workability preference (at the environment-individual interaction level), and landscape accessibility preference (at the cognitive level of advantages and disadvantages of natural environment). Therefore, the impact of the green exposure index on activity behavior was first based on the intermediate function of spatial perception which only affected activity frequency without affecting activity time, further demonstrating that spatial perception, which formed the basis of the instantaneous emotional response process, may stimulate activity motivation by fast responding to the internal perception level so as to affect personal decision-making when conducting activities but is unable to affect the time-space continuity of activity behavior.

### 4.4. The spatial pattern of the community green spatial perception

In recent years, the research method of combining the spatial metrics of the green exposure index with micro evaluation of spatial perception has been regarded as a new method that forms a qualitative research framework based on quantitative analysis of urban green space quality. Through empirical analysis, it is found that a large number of influencing factors are mixed in issues concerning urban green space, physical activity behavior, and health (37), and the potential internal association as well as the spatial pattern of these issues need to be considered within places of specific scales (25). Therefore, the impact of community green space quality on spatial perception can be adjusted to some extent by identifying residence-based buffer areas. During the investigation process, it was commonly suggested by the research subjects and investigators that the greening quality and public facilities of the community with security housing represented by the Xinglongyuan community were obviously poorer than those of communities with commercial housing and units, but the proportion of importance of the eight perception measuring items and the mean value of spatial perception evaluation (the results were not shown) were significantly higher than those of the other communities. Concerning the reason for the above findings, on the one hand, in the Xinglongyuan community, as a community with security housing, the green exposure index remained at the medium level. On the other hand, most communities with security housing covered a floor area of <10,000 m^2^, which meant that the buffer area covered an area of <100 m. Therefore, a smaller floor area may have a more direct impact on people's space experience and activity behavior in the community green space with activity boundaries.

### 4.5. Limitations

In this study, the activity behavior survey mainly focused on the activity time and frequency of middle-aged and older adults in the community, and other factors of activity behavior were not explained. During the investigation, it was found that some older adults' evaluation of activity behavior may be inaccurate. In addition, the survey respondents were generally middle-aged and older adults aged 50–70 years, and the research conclusions may have a group phenomenon. In future research, it is necessary to improve the research scheme based on different age groups.

## 5. Conclusion

Green exposure index, spatial perception, and activity behavior have an internal influence relationship, and the effect of the green exposure index on activity behavior is based on the intermediate role of spatial perception. Spatial perception, as the basis of an instantaneous emotional response process, may stimulate activity motivation through the rapid response of the internal perception level and influence individuals' decisions to implement activity behavior. It is worth noting that the influence of the green exposure index on spatial perception and activity behavior is limited by activity area and activity boundary.

## Data availability statement

The original contributions presented in the study are included in the article/supplementary material, further inquiries can be directed to the corresponding authors.

## Author contributions

LZ: conceptualization, methodology, software, data curation, writing—original draft preparation, and writing—review and editing. YZ: methodology and writing—review and editing. RD: methodology, writing—review and editing, supervision, and funding acquisition. ZW and JS: conceptualization and supervision. All authors contributed to the article and approved the submitted version.
